# Mixed Disulfide Formation at Cys141 Leads to Apparent Unidirectional Attenuation of *Aspergillus niger* NADP-Glutamate Dehydrogenase Activity

**DOI:** 10.1371/journal.pone.0101662

**Published:** 2014-07-02

**Authors:** Adhish S. Walvekar, Rajarshi Choudhury, Narayan S. Punekar

**Affiliations:** Department of Biosciences and Bioengineering, Indian Institute of Technology Bombay, Mumbai, Maharashtra, India; College of Medicine, University of South Florida, United States of America

## Abstract

NADP-Glutamate dehydrogenase from *Aspergillus niger* (AnGDH) exhibits sigmoid 2-oxoglutarate saturation. Incubation with 2-hydroxyethyl disulfide (2-HED, the disulfide of 2-mercaptoethanol) resulted in preferential attenuation of AnGDH reductive amination (forward) activity but with a negligible effect on oxidative deamination (reverse) activity, when monitored in the described standard assay. Such a disulfide modified AnGDH displaying less than 1.0% forward reaction rate could be isolated after 2-HED treatment. This unique forward inhibited GDH form (FIGDH), resembling a hypothetical ‘one-way’ active enzyme, was characterized. Kinetics of 2-HED mediated inhibition and protein thiol titrations suggested that a single thiol group is modified in FIGDH. Two site-directed cysteine mutants, C141S and C415S, were constructed to identify the relevant thiol in FIGDH. The forward activity of C141S alone was insensitive to 2-HED, implicating Cys141 in FIGDH formation. It was observed that FIGDH displayed maximal reaction rate only after a pre-incubation with 2-oxoglutarate and NADPH. In addition, compared to the native enzyme, FIGDH showed a four fold increase in K_0.5_ for 2-oxoglutarate and a two fold increase in the Michaelis constants for ammonium and NADPH. With no change in the GDH reaction equilibrium constant, the FIGDH catalyzed rate of approach to equilibrium from reductive amination side was sluggish. Altered kinetic properties of FIGDH at least partly account for the observed apparent loss of forward activity when monitored under defined assay conditions. In sum, although Cys141 is catalytically not essential, its covalent modification provides a striking example of converting the biosynthetic AnGDH into a catabolic enzyme.

## Introduction

Glutamate dehydrogenase (GDH) catalyzes reversible reductive amination of 2-oxoglutarate to form l-glutamate using NAD(P) as a cofactor [1]. A variety of methods are employed to trace the amino acid residues forming the active and the regulatory site(s). A range of GDH activity responses have been recorded upon thiol modification, that depend on the nature of the reagent and the target site. There are no conserved cysteines across GDH sequences from various domains of life. The reactive cysteines in GDHs from diverse sources have been identified with the use of affinity labels [2]–[4] and thiol specific reagents [5]–[9]. Characterization of site-directed mutants has also helped to understand the role of the target thiols in different GDHs [2], [8], [9]. Cys323 in human GDH isozymes (hGDH1 and hGDH2) is so far the only reactive cysteine identified as important for catalysis [9]. Another cysteine residue, Cys119, is shown to be ADP-ribosylated in both these isozymes. ADP-ribosylation affects forward as well as reverse activity of the enzyme. The physiological significance of this modification is being explored currently [10].

Pure preparations of NADP-GDH of *Aspergillus niger* (AnGDH), upon storage in the presence of 2-mercaptoethanol at 4°C, selectively lost the reductive amination (forward) activity as monitored in the standard assay. We refer to this phenomenon as ‘forward inhibition’ throughout the manuscript. Preferential inhibition of enzyme activity in one direction has been discussed earlier [11] and is reported for few enzymes [12]–[17]. So far, sucrose synthase provides the lone example of a disulfide mediated reversible, unidirectional enzyme inhibition [16]. AnGDH differs in kinetic and biochemical aspects from its *Aspergillus terreus* homolog (AtGDH) [18], [19]. Despite substantial sequence similarity and five conserved cysteine residues, the two enzymes are differentially reactive towards 2-hydroxyethyl disulfide (2-HED; the disulfide of 2-mercaptoethanol). Only AnGDH is sensitive to the disulfide treatment and the associated preferential loss in its forward velocity is noteworthy. Such an enzyme form of AnGDH could be isolated (and termed ‘FIGDH’ for ‘Forward Inhibited GDH’). We report the characterization of this practically one-way active enzyme and have identified the cysteine residue modified in FIGDH, by site-directed mutagenesis.

## Materials and Methods

### Materials

All the chemicals were of analytical grade and from either Sigma-Aldrich Chemicals Pvt. Ltd. (St. Louis, MO, USA) or local suppliers. Sephadex G-25 was from GE Healthcare (formerly, Pharmacia Fine Chemicals, Uppsala, Sweden).

2-HED was either purchased from Sigma-Aldrich Chemicals Pvt. Ltd. (St. Louis, MO, USA) or prepared by two methods. In the method developed in-house, H_2_O_2_ (0.5 M) was added drop-wise to 100 mM 2-mercaptoethanol. Aliquots were checked for thiol content by treatment with 5,5'-dithiobis-(2-nitrobenzoic acid) (DTNB) [20]. Excess H_2_O_2_ was destroyed by incubation with catalase (650 Units) at 37°C for 1 h. The sample was then filtered through a 3 kDa cut-off ultra-filtration membrane (YM 3, Millipore, Bedford, MA, USA) to remove catalase. In the second method, 2-HED was prepared according to Leung and Hoffmann [21] but with minor modifications. 2-Mercaptoethanol solution was prepared in 0.1 M carbonate buffer (pH 10.4) and 10 µM EDTA. The concentration of the product formed was calculated from its absorbance at 248 nm using an extinction coefficient [22] of 324 M^−1^cm^−1^.

### Strains, media and growth conditions

*A. niger* NCIM 565 and *A. terreus* NCIM 656 were obtained from National Collection of Industrial Microorganisms at National Chemical Laboratories, Pune, India and maintained as mentioned elsewhere [18]. *Escherichia coli* strains (XL1-Blue and BL21(DE3)) were grown on Luria-Bertani broth and maintained as glycerol stocks.

### Heterologous expression of *A. niger* NADP-GDH and its site-directed mutants

The *gdhA* gene (encoding NADP-GDH) from *A. niger* strain ATCC 1015 (http://genome.jgi.doe.gov/Aspni5/Aspni5.home.html; scaffold 13, contig 7, nucleotides 959641-961142) contains two introns. Genomic amplification of NADP-GDH gene from *A. niger* NCIM 565 and its sequencing confirmed the presence of two introns (GenBank ID: ACJ03788). The cDNA was amplified for heterologous expression and also to generate site-directed mutants. Total mRNA was prepared from *A. niger* mycelia using Qiagen RNeasy plant mini kit and following primers were used (restriction enzyme recognition sites are shaded while base changes made to generate these restriction sites are underlined): forward, 5′TCGAATTCCATATGTCTAACCTTCCTCAGCAG3′ and reverse, 5′GTTTGAAGCTTTCCGCATTTACCACCAGTC3′. The cDNA obtained was cloned into pBlueScript as its *Eco*RI-*Hin*dIII fragment. This plasmid (named pBScGDH) was characterized and sequenced. The cDNA was then subcloned into pET43.1(b) between *Nde*I and *Hin*dIII sites. This final construct, named as pETAnGDH, was used to transform *E. coli* BL21(DE3) cells for over-expression of recombinant AnGDH (without a tag).

C141S and C415S site-directed mutants were generated by PCR amplification of pETAnGDH as the template using suitably mismatched primers (base changes are underlined and the altered codons are in bold):

For C141S: forward, 5′CCTTCATGACCGAGCTC**TCC**AAGCACATCG3′

reverse, 5′CGATGTGCTT**GGA**GAGCTCGGTCATGAAGG3′

For C415S: forward, 5′CATCATGCGCGAC**TCC**TTCAAGAACGG3′

reverse, 5′CGTTCTTGAA**GGA**GTCGCGCATGATGTCC3′

After amplification, template pETAnGDH was digested using *Dpn*I and the PCR product was transformed in *E. coli* BL21(DE3). All the amplifications and desired mutations were confirmed by DNA sequencing.

### Enzyme preparations

NADP-GDHs from *A. niger* and *A. terreus* were extracted and purified as reported previously [18], [19]. The purification buffer (pH 7.5) contained 20 mM potassium phosphate buffer, 4.0 mM 2-mercaptoethanol and 1.0 mM EDTA. To avoid thiol interference, whenever appropriate, 2-mercaptoethanol was omitted from the buffer. For over-expression of AnGDH, C141S and C415S, *E. coli* BL21(DE3) cells transformed with respective plasmids were grown to mid-log phase and then induced with isopropyl β-D-1-thiogalactopyranoside (0.3 mM) at 25°C for 12 h. The cells were harvested by centrifugation (10,000 g, 20 min at 4°C), resuspended in purification buffer and then disrupted by sonication. Lysed cells were centrifuged (10,000 g, 20 min at 4°C) and supernatant was used as the ‘crude extract’. The subsequent steps were identical as reported for native AnGDH purification [19]. *E. coli* BL21(DE3) also has its own NADP-GDH [23]. A comparison of NADP-GDH specific activities from crude extracts and purified enzyme fractions of untransformed and transformed BL21(DE3) cells and electrophoretic analysis showed that endogenous *E. coli* enzyme contribution to the final purified preparations was less than 0.5% (also monitored by activity staining), if any.

Protein was estimated using dye binding method [24] with bovine serum albumin as a reference. Purity of the enzyme preparation was checked by native polyacrylamide gel electrophoresis (PAGE) [25] and subsequent staining with Coomassie Blue R-250.

NADP-GDH activity was measured by following the change in absorbance at 340 nm in the linear region of the reaction [19]. Reductive amination (forward activity) was determined in a reaction mixture (1.0 ml) containing 100 mM Tris-HCl buffer (pH 8.0), 10 mM ammonium chloride, 10 mM 2-oxoglutarate (pH adjusted to 8.0) and 0.1 mM NADPH. The reaction was routinely started by the addition of NADPH. This standard assay was suitably modified to kinetically characterize FIGDH forward activity (see below). Oxidative deamination (reverse activity) was determined in a reaction mixture (1.0 ml) containing 100 mM Tris-HCl buffer (pH 9.3), 0.4 mM NADP^+^ and 100 mM l-glutamate (pH adjusted to 9.3). Here, the reaction was started by the addition of NADP^+^. One enzyme unit corresponds to the amount of enzyme required to reduce/oxidize one µmol of NADP^+^/NADPH min^-1^ at room temperature.

Typically, pure AnGDH preparations had a specific activity of 150 U mg^−1^ protein and 65 U mg^−1^ protein in the standard forward and reverse assays, respectively. The corresponding specific activities for AtGDH were 40 U mg^−1^ protein and 15 U mg^−1^ protein, respectively.

### Incubation of NADP-GDH with 2-hydroxyethyl disulfide

Different NADP-GDH forms (namely, AnGDH, AtGDH, C141S and C415S) were incubated with 2-HED (2 mM) at 37°C. Aliquots were withdrawn at different time intervals and forward and reverse rates were measured using the respective standard assays. To obtain the FIGDH form, AnGDH was incubated with 2-HED till the preparation showed less than one percent forward velocity with respect to the control. The reaction mixture was immediately buffer exchanged using a 10 kDa cut-off ultra-filtration membrane (YM 15, Millipore, Bedford, MA, USA) with purification buffer lacking 2-mercaptoethanol, to stop further reaction.

### Reactivation of FIGDH with different thiols

FIGDH was incubated with different thiols (20 mM each; 10 mM in the case of dithiothreitol (DTT)) at 37°C and aliquots withdrawn at different time intervals to test for forward and reverse rates using the respective standard assays. All thiol stock solutions were prepared in purification buffer (but without 2-mercaptoethanol) and the pH was adjusted to 7.5 before use.

### Determination of number of thiols modified in FIGDH

a) Activity based approach: AnGDH was incubated with various fixed concentrations of 2-HED at 37°C; aliquots were withdrawn at different time intervals and tested for the loss of forward reaction rate. This disulfide modification time course data essentially followed pseudo-first order kinetics. The slope of the plot ‘log (percent activity remaining) versus incubation time’ gave k_app_ (apparent inactivation constant) values at respective 2-HED concentrations. The number of thiols modified was inferred (as the slope) [26] from the replot of log k_app_ versus log [2-HED]. b) Protein based approach: Free thiols from AnGDH and FIGDH preparations were estimated using DTNB [20]. The enzyme (at 1.0–2.0 µM of the monomer; calculated from protein concentration and subunit composition) was denatured in guanidine-HCl (1.8 M, final concentration). Denatured preparations were subsequently treated with excess DTNB (500 µM in 0.1 M sodium phosphate buffer, pH 7.2) and absorbance measured at 412 nm. The number of free thiols was calculated from the 2-nitro-5-thiobenzoate formed (ε_412 nm_  = 13,600 M^−1^ cm^−1^).

### Kinetic characterization of FIGDH

High concentrations of 2-oxoglutarate absorb strongly at 340 nm (A_340 nm_ of 1.0 at 50 mM). Therefore, FIGDH forward activity was measured at 370 nm (and a factor of 2.29 applied to calculate the NADPH consumed) where the 2-oxoglutarate absorbance contribution was minimal. The assay also incorporated a 15 min pre-incubation with 2-oxoglutarate and NADPH to attain maximal FIGDH initial velocity (pre-incubation with these two substrates abolished the lag displayed in the FIGDH time course and allowed clean initial rate measurements; see Results section). FIGDH required relatively higher concentrations of 2-oxoglutarate and ammonium for saturation; suitable additions of NaCl were made to adjust/maintain the ionic strength in the assay. With the exception of the substrate being varied, the assay mixture (1.0 ml) for forward reaction typically contained 100 mM Tris-HCl buffer (pH 8.0), 100 mM ammonium chloride, 50 mM 2-oxoglutarate (pH adjusted to 8.0) and 0.2 mM NADPH. The reaction was always initiated with ammonium addition. However, the assay conditions for FIGDH reverse activity measurements were identical to those for the native enzyme (mentioned above).

### Determination of equilibrium constant

AnGDH or FIGDH was incubated with fixed concentrations of all the forward and reverse substrates while only [NADP^+^] was varied. FIGDH showed very poor forward reaction rates with substrate concentrations used in the standard AnGDH assay. Choice of higher substrate concentrations gave reliable measures of activity in both the directions, for the two enzyme forms. The choice of substrate concentrations in the experiment was such that net forward and reverse activities were observed at the lowest and highest [NADP^+^], respectively. For GDH reaction, the ‘mass action ratio’ (Γ) expression [27], was used. The equilibrium constant (K_eq_) numerically equals Γ at a concentration of NADP^+^ where the enzyme shows no net forward or reverse reaction rate. The K_eq_ was thus calculated from the rate readings of 4–5 points near and on both sides of equilibrium (the point where ΔA_340 nm_ min^−1^ is zero). Linear or non-linear data fits were used as appropriate to fit the data.

### Circular dichroism

The CD spectra were recorded between 190–260 nm with Jasco J-810 CD polarimeter with the following instrument settings: response time, 1 s; scanning speed, 50 nm min^-1^; temperature, 25°C.

### Gel filtration

HiLoad 16/60 Superdex 200 preparatory grade (GE Healthcare, Amersham) column was calibrated using a set of protein molecular weight markers (MWGF1000 kit; Sigma-Aldrich Chemicals, St. Louis, Missouri, USA). The column was equilibrated and then developed (1.0 ml min^−1^) at room temperature with the purification buffer containing 0.15 M NaCl. The elution volume (V_e_) of blue dextran (2000 kDa) was taken as void volume (V_o_). Elution coefficient (K_av_) [28] was determined using the formula: K_av_  =  (V_e_-V_o_)/(V_t_-V_o_), where V_t_ is the total column volume (120 ml).

### Statistical analysis of data

The data presented are typical of at least three independent experiments with replicates. SigmaPlot 12.0 was used for kinetic data analysis. Experimentally determined values are presented as points and the lines represent best fits. The error bars in figure legends correspond to mean ± standard deviation.

### Bioinformatic analysis

ClustalW [29] was used for sequence comparisons. Homology modeling of AnGDH and AtGDH was done using I-TASSER (http://zhanglab.ccmb.med.umich.edu/I-TASSER/) with *E. coli* NADP-GDH as the template. The model development for the hexamer and distance measurements were done using WinCoot [30] version 0.7. The PyMOL molecular graphics system (version 1.3 Schrödinger, LLC) was used for generating active site structures.

## Results

### Apparent one-way inactivation of *A. niger* NADP-GDH

**Figure 1:**
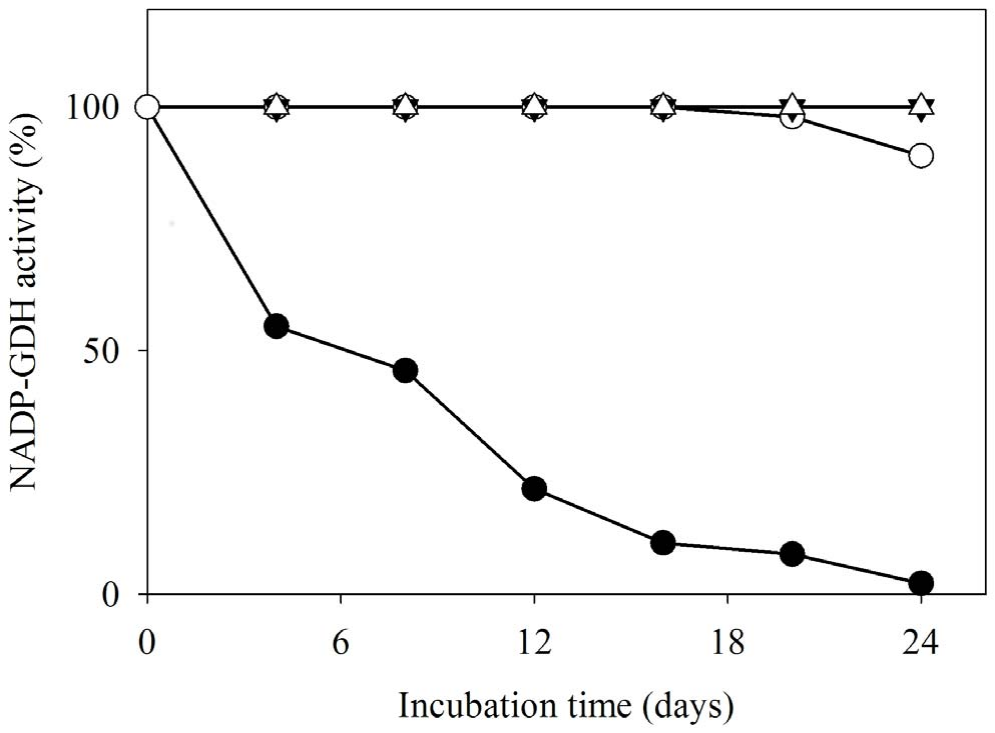
Selective attenuation of AnGDH forward activity upon storage. Forward (filled symbols) and reverse (open symbols) activities were monitored as a function of time of storage at 4°C in 2-mercaptoethanol containing buffer. In these standard NADP-GDH assays, 4.0 U of AnGDH and 3.5 U of AtGDH were used. Comparative data for purified AnGDH (•,○) and AtGDH (▾,Δ) are shown.

Pure preparations of AnGDH when stored at 4°C showed time-dependent loss of forward activity (as monitored by the standard assay) while retaining full activity in the reverse direction. The forward to reverse activity ratio for AnGDH (in the respective standard assays) changed from 2.5∶1 to 0.01∶1. However, such an effect was not noted for AtGDH activity (Figure 1). Several possibilities were explored to address the apparent ‘one-way’ inactivation of AnGDH upon storage; thiol modification was one of them. AnGDH purified in a 2-mercaptoethanol-free buffer retained its full activity in both the directions even after a month of storage. This led us to hypothesize an air oxidation of 2-mercaptoethanol to form 2-HED; the disulfide formed then reacts with enzyme thiol(s) thereby affecting the forward activity. AnGDH (purified in the purification buffer lacking 2-mercaptoethanol) was incubated with 2-HED (2.0 mM, 37°C) to test this proposal. The enzyme forward velocity declined rapidly (within 3 h) in a time-dependent manner, confirming the participation of thiol modification. A slow loss of enzyme reverse velocity was also observed upon extended treatment with 2-HED (see below). AnGDH purified from *A. niger* mycelia as well as the recombinant enzyme (heterologously expressed in *E. coli*) showed a similar pattern of inhibition when incubated with 2-HED. Since the AnGDH obtained either way was indistinguishable with respect to other kinetic and physical properties (Table 1), the enzyme expressed in *E. coli* was routinely used in further experiments.

**Table 1:** Summary of kinetic parameters of different AnGDH forms.

	K_m_ (mM) [*n*_H_]^a^					V_max_ (U mg^−1^ protein)
Enzyme	2-Oxoglutarate	NH_4_^+^	NADPH	L-Glutamate	NADP^+^	Forward	Reverse
AnGDH (mycelial)^b^	4.78 [3.2]	1.05	0.011	34.6	0.017	111.1	52.7
AnGDH (recombinant)	5.70 [3.5]	1.1	0.023	30.9	0.028	193.7	78.7
C141S	4.00 [2.3]	1.2	ND	28.6	ND	265.4	108.2
C415S	15.50 [4.9]	Biphasic^c^	ND	36.8	ND	198.7	104.7
FIGDH^d^	25.10 [2.4]	2.6	0.044	49.8 [1.9]	0.022	150.7^e^	112.3

a K_0.5_ is reported for sigmoid saturations with respective *n*_H_ values in square brackets; ^b^Data as reported previously [19]; ^c^Details to be communicated separately; ^d^For assay details see Materials and Methods; ^e^Velocity measured under standard forward assay was 0.7 U mg^−1^ protein; ND: not determined.

To ascertain the generality of disulfide-mediated forward inhibition, the effect of oxidized glutathione (GSSG) and cystine on AnGDH activity was tested (Table 2). The forward and reverse activities of the enzyme were not affected by GSSG. Cystine, on the other hand, strongly and selectively inhibited the forward reaction in a manner similar to 2-HED. The enzyme form having negligible forward (less than 1.0% of the control) but complete reverse activity (almost 100% of the control) was of interest. This FIGDH form was prepared by 2-HED treatment and isolated, as described in Materials and Methods.

**Table 2:** Effect of different disulfides on forward and reverse activities of AnGDH.

Disulfide (5.0 mM)	Residual activity (%)	
	Forward	Reverse
2-HED	0.8±0.2	100.4±5.8
GSSG	100.7±7.3	100.0±2.3
Cystine (saturated^a^)	8.3±1.9	101.0±4.8

a Saturating concentration in water corresponds to 0.47 mM at 25°C [56].

AnGDH (1.0 U) was incubated for 1 h with each disulfide at 37°C and the residual activity was measured under standard assay conditions. The AnGDH activity remaining without any disulfide treatment after 1 h incubation is taken as control (100%). Mean ± standard deviation (n≥2) are presented.

### Reactivation of FIGDH

**Figure 2:**
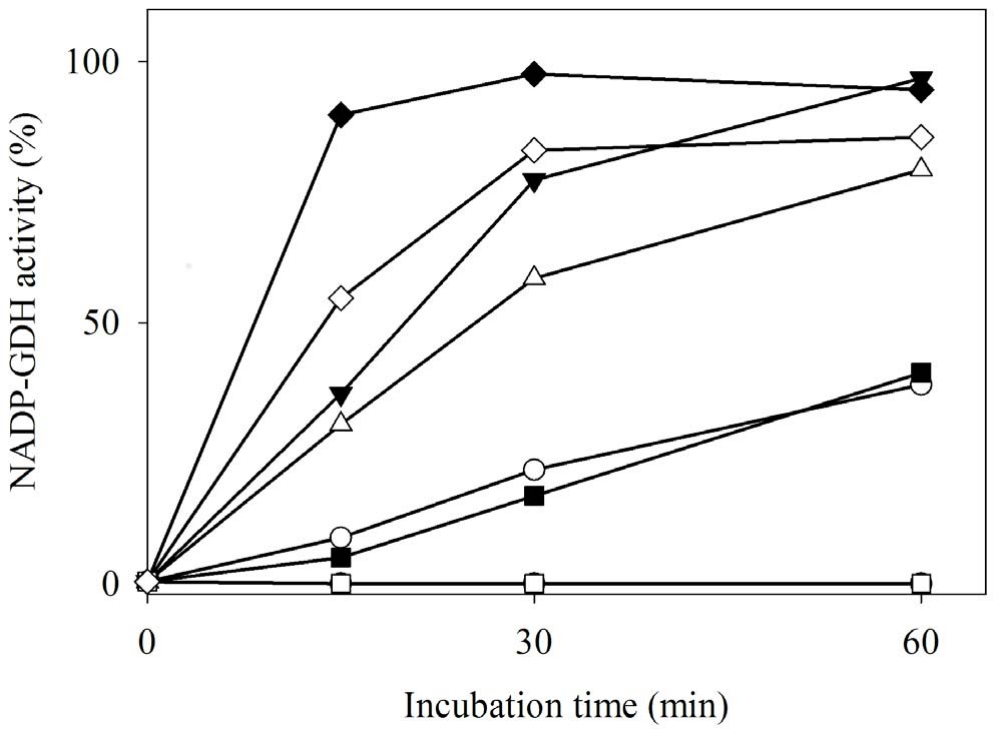
Reactivation of FIGDH by incubation with different thiols. FIGDH (4.4 µg pure protein in 400 µl) was incubated at 37°C with different thiols (•, no thiol; ○, 2-mercaptoethanol; ▾, DTT; Δ, cysteine; ▪, GSH; □, thioglycolate; ♦, methyl thioglycolate and ◊, cysteamine) and 20 µl aliquots were withdrawn to monitor forward activity by the standard assay. The concentration of each thiol was 20 mM except in case of DTT (10 mM; to maintain equivalence of -SH groups).

Different thiols were tested for their ability to reactivate FIGDH (Figure 2). Neutral or positively charged thiols were effective in restoring the forward activity, albeit with varying efficacy. Reduced glutathione (GSH) and cysteine (physiologically relevant thiols) were also effective in this reactivation. Interestingly, methyl thioglycolate, but not thioglycolic acid, was found to reactivate FIGDH. The presence of negative charge may render thioglycolate inaccessible to the site of thiol modification.

### Only one thiol is modified in FIGDH

**Figure 3:**
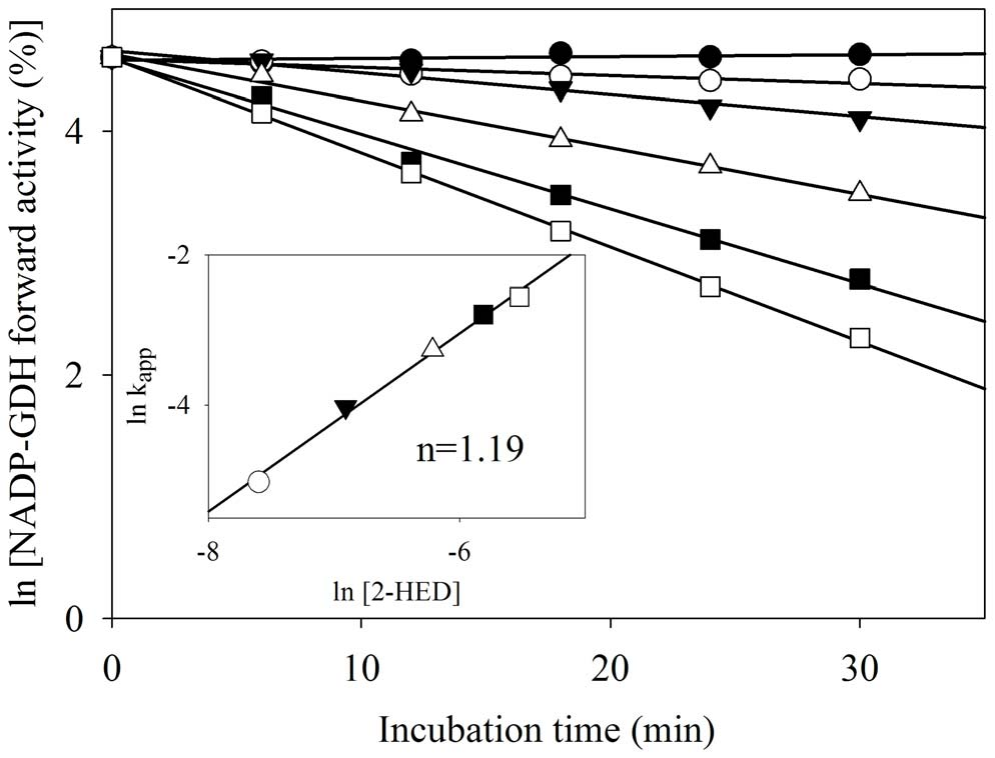
Kinetics of 2-HED mediated inactivation of AnGDH forward activity. AnGDH (50 µg pure protein in 200 µl) was treated with different concentrations of 2-HED (•, 0 mM; ○, 0.5 mM; ▾, 1.0 mM; Δ, 2.0 mM; ▪, 3.0 mM and □, 4.0 mM). Aliquots (20 µl) were withdrawn (and diluted suitably) at time points indicated, to monitor the residual activity by the standard assay. Inset: log-log plot of k_app_ versus 2-HED concentrations (with corresponding symbols) to obtain the number of catalytically important thiols modified.

AnGDH has five cysteine residues in its primary sequence (these five residues are also conserved in AtGDH) [18]. The number of thiols modified by 2-HED, and hence involved in forward inhibition, was determined. In an activity based approach, AnGDH was treated with different concentrations of 2-HED and its forward activity was followed with time (Figure 3). A plot of log k_app_ versus log [2-HED] (with slope  = 1.19 and second order inactivation rate constant  = 56.2 M^−1^ min^−1^; Figure 3, inset) suggested the involvement of one reactive thiol per monomer in 2-HED mediated forward inhibition. Such an activity based approach would miss the catalytically silent thiols. Therefore, the number of free thiols in AnGDH and FIGDH was estimated by direct DTNB titrations, under denaturing conditions. Results indicated the absence of disulfide groups in AnGDH and masking of a single thiol per monomer in FIGDH (Table 3). By Matrix Assisted Laser Desorption/Ionization-Time of Flight mass spectrometry, AnGDH showed M_r_ of 49227±76 (n = 6) whereas FIGDH showed M_r_ of 49327±95 (n = 5). The observed mass shift suggested an average of one thiol modified per monomer of FIGDH.

**Table 3:** The number of free thiols found in AnGDH and FIGDH.

Enzyme	Free thiol groups per monomer
AnGDH	5.19±0.49
FIGDH	4.09±0.62

The DTNB titration data presented are for enzymes denatured in 1.8 M guanidine-HCl. The results were analyzed using paired *t*-test, mean ± standard deviation (n = 3, P = 0.016) are shown.

### Cys141 modification leads to attenuation of *A. niger* NADP-GDH forward activity

**Figure 4:**
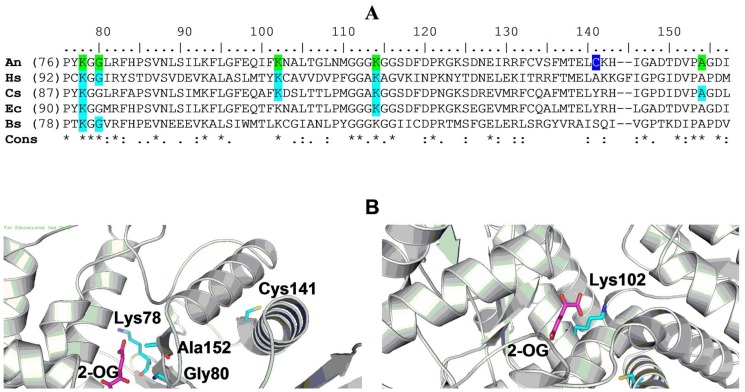
Location of Cys141 and Cys415 in the context of active site residues in AnGDH. **A)** The GDH sequences (from UniProtKB database) from *A. niger* (An; B6V7E4), *Homo sapiens* (Hs; P00367), *C. symbiosum* (Cs; P24295), *E. coli* (Ec; P00370) and *Bacillus subtilis* (Bs; P39633) were compared (ClustalW). Residues implicated in glutamate binding (for GDHs where experimental evidence exists; shaded cyan) and the corresponding AnGDH residues (shaded green) are indicated. Cys141 is highlighted (in blue). **B)** AnGDH homology model showing Cys141 (left panel) and Cys415 (right panel) in the context of the enzyme active site. 2-OG: 2-Oxoglutarate.

Two site-directed mutants (namely C141S and C415S) were generated to uncover the thiol involved in forward inhibition. Kinetic characterization of FIGDH showed that substrates occupying glutamate-binding domain display significantly altered interaction (see below). Hence Cys residues at positions 338 and 349 in the dinucleotide-binding domain were not considered. Cys133 was also ignored because *E. coli* NADP-GDH is not sensitive to 2-HED treatment (data not shown) despite the fact that it is the only cysteine conserved between *E. coli* and *A. niger* enzymes (Figure 4A). For these reasons, Cys141 was chosen as a candidate for site-directed mutagenesis. Cys415 was selected because, according to the homology model (see Materials and Methods), it was predicted to be solvent accessible and lies in a hinge helix between the glutamate- and dinucleotide-binding domains.

Both C141S and C415S mutant proteins were expressed in *E. coli* BL21(DE3) and purified to homogeneity. The specific activities of C141S and the native enzyme were comparable (Table 1), suggesting that Cys141 may not be essential for catalysis. Although C415S was catalytically active, it had higher K_0.5_ for 2-oxoglutarate (15.5 mM compared to 5.7 mM in case of AnGDH; Table 1) and displayed biphasic ammonium saturation (manuscript under preparation). Circular dichroism spectra, native PAGE and gel filtration analyses of these mutant forms indicated no change in gross secondary structure and oligomeric state for the two mutants (Figure S1).

**Figure 5:**
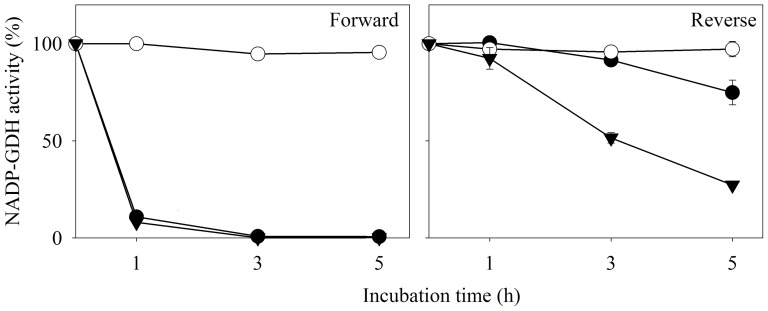
Effect of 2-HED on forward and reverse activities of AnGDH and its two C→S mutants. AnGDH enzyme forms (•, AnGDH; ○, C141S and ▾, C415S) were separately incubated with 2-HED (2.0 mM, at 37°C) and their forward (left panel) and reverse (right panel) activities were monitored in the standard assay. All incubations contained 5–7 µg of respective pure enzyme protein. The forward assay for C415S contained 30 mM 2-oxoglutarate (instead of 10 mM used in the standard assay) to account for its higher K_0.5_.

Both C141S and C415S proteins were treated with 2-HED (2.0 mM, 37°C) and their initial velocities in the two directions were monitored, as a function of time (Figure 5). C141S was resistant to the disulfide treatment while C415S lost its forward activity in a manner very similar to the native enzyme. Both forward and reverse velocities of C141S were unaffected even after incubation with 10 mM 2-HED for 5 h (not shown). However, the C141S substrate saturation patterns and kinetic constants (Table 1) were similar to those of the native enzyme. Taken together, 2-HED inhibition kinetics, DTNB titration and physico-chemical analysis of AnGDH, FIGDH, C141S and C415S strongly implicate Cys141 in the phenomenon of forward inhibition.

### Characterization of FIGDH. A) Kinetic features of FIGDH

In the standard assay (with 10 mM ammonium chloride, 10 mM 2-oxoglutarate and 0.1 mM NADPH), very little forward reaction rate was displayed by FIGDH. This marked loss in activity could be a reflection of decreased affinity for a substrate(s) and/or an altered pH optimum for the forward reaction. The first possibility was tested by raising concentration of substrates in the assay. FIGDH displayed significantly increased forward velocity when higher concentrations of 2-oxoglutarate and ammonium were used. However, the maximal rate was attained after an initial lag. This lag disappeared when the enzyme was pre-incubated with 2-oxoglutarate and NADPH before starting the reaction (by ammonium). Also, the FIGDH forward velocity increased 3-4 fold upon pre-incubation with 2-oxoglutarate and NADPH. Hence the initial velocity measurements were routinely made after a pre-incubation of 15 min and by starting the reaction with ammonium (see Materials and Methods for details). FIGDH exhibited sigmoid 2-oxoglutarate saturation with four-fold higher K_0.5_ (Table 1). The Michaelis constants for ammonium and NADPH were also more than two fold higher for FIGDH when compared to those of AnGDH (Table 1). The changes in the FIGDH pH profiles, if any, were also monitored. The FIGDH pH optima (both forward and reverse reactions) were not significantly different from those of AnGDH (Figure S2).

Since FIGDH and AnGDH displayed comparable velocities in the standard reverse assay, detailed kinetic characterization in this direction was straightforward. While NADP^+^ saturation was typically Michaelian, l-glutamate saturation of FIGDH was sigmoid (*n*_H_  =  1.9). On the whole, the kinetic parameters (summarized in Table 1) suggest significantly altered substrate interactions with glutamate-binding domain of FIGDH.

### B) FIGDH catalyzed rate of approach to equilibrium is sluggish from reductive amination direction

**Figure 6:**
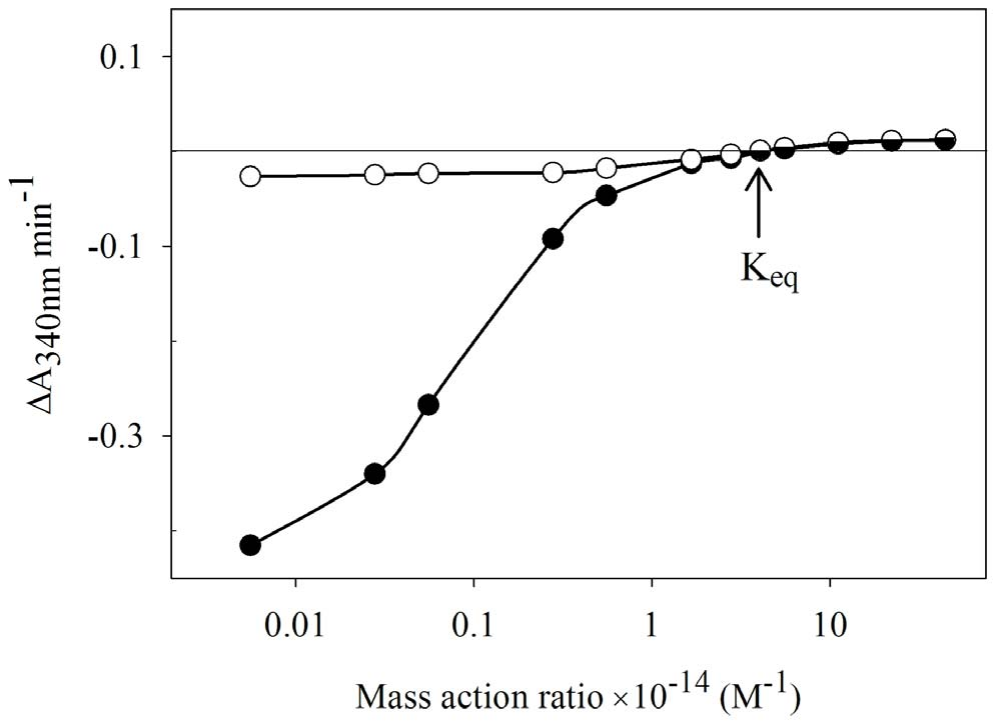
Determination of equilibrium constant for GDH reaction catalyzed by AnGDH and FIGDH. The two enzyme forms (•, AnGDH; ○, FIGDH) were separately incubated with fixed concentration of all substrates and the activity was monitored by varying only [NADP^+^]. The reaction mixture (final pH adjusted to 8.0) contained 100 mM Tris-HCl buffer, 20 mM 2-oxoglutarate, 100 mM l-glutamate, 5.0 mM ammonium chloride and 100 µM NADPH. NADP^+^ concentration was varied from 1.0 µM to 8.0 mM. Mass action ratio (Γ) was calculated as mentioned in Materials and Methods. In order to account for changing net forward and reverse rates, different enzyme amounts were used; the observed reaction rates were accordingly normalized for equal amount of protein in both the cases. Note that negative ΔA_340 nm_ values mean net forward activity while positive ΔA_340 nm_ values mean net reverse activity. The point where no net ΔA_340 nm_ occurs corresponds to K_eq_ (indicated by the arrow).

The apparent inhibition of forward activity (as monitored by the standard assay) was due to altered kinetic properties of FIGDH. Since the attenuation of FIGDH activity was largely unidirectional, it was useful to evaluate the rate of approach to equilibrium from the two directions. A plot of net reaction rate versus mass action ratio (Γ) was used to determine the K_eq_ (see Materials and Methods); the equilibrium constant for the GDH reaction remained unchanged (∼4×10^14^ M^−1^) regardless of whether AnGDH or FIGDH was the catalyst (Figure 6). The rates of approach towards equilibrium were similar for both the enzyme forms when advancing from the higher NADP^+^ concentrations (i.e. in the reverse direction). But for AnGDH the curve was steeper than that for FIGDH when advancing from lower [NADP^+^] side. 2-HED modification of Cys141 thus makes AnGDH a less effective catalyst in the direction of l-glutamate synthesis with no discernible difference in the direction of l-glutamate catabolism.

## Discussion

Earlier work has shown that AnGDH and AtGDH differ in their 2-oxoglutarate saturation and chymotrypsin cleavage pattern [18], [19]. These differences are remarkable considering that the two enzymes share 88% identity and 93% similarity at the amino acid level. Here we report that only AnGDH (and not AtGDH) displays storage-dependent inhibition of forward activity (in the defined standard assay) without much effect on its ability to catalyze the reverse reaction (Figure 1). The mechanistic reasons for this remarkable apparent unidirectional attenuation of AnGDH activity were explored. The spontaneous deamidation of asparaginyl residues of AnGDH, as observed with *Saccharomyces cerevisiae* GDH3p [31] and *Clostridium symbiosum* D165N GDH mutant [32], was a possibility. This was ruled out as AnGDH did not show characteristic features of deamidation namely, i) a shift in mobility on native PAGE and ii) an increased instability at higher pH (data not shown). 2-Mercaptoethanol (a component of the purification buffer) is known to form 2-HED upon air oxidation [33]. The native enzyme did not show any loss of forward activity when purified in a buffer lacking 2-mercaptoethanol, suggesting the involvement of 2-HED formed from 2-mercaptoethanol. This prediction was confirmed when FIGDH could be prepared directly by incubating AnGDH with preformed 2-HED (Figure 5).

Preferential attenuation of AnGDH forward activity by 2-HED and reactivation with different thiols (Figure 2) pointed to the formation of a mixed disulfide. 2-HED and other disulfides are known to react with exposed free protein thiols to form mixed disulfides [16], [34]–[38]. Although such modifications are reversible, low molecular weight thiols differ in their ability to regenerate the native protein. Such reactivation experiments actually probe the accessibility of added thiol to the mixed disulfide site. Efficacy of FIGDH reactivation followed the order (Figure 2): methyl thioglycolate, DTT, cysteamine, cysteine >2-mercaptoethanol, GSH >> thioglycolic acid. It appears that carboxylate group hinders the accessibility of thioglycolic acid to the disulfide site. Masking this carboxylate group (namely, methyl thioglycolate) rendered the thiol very effective in reactivation. Small, neutral or positively charged thiols were able to easily access the site of modification in FIGDH. In an earlier study, instead of using different thiols to reactivate the 2-HED inactivated enzyme, various thiol disulfides were tested for their ability to inactivate aldehyde dehydrogenase [34]. Interestingly, negatively charged or bulky disulfides were found less effective than small, uncharged disulfides. 2-Mercaptoethanol (and its disulfide) seems to have better access to thiol groups due to its smaller size. The inhibition with disulfides like 2-HED and cystine (Table 2) and subsequent reactivation with different thiols (Figure 2), highlight the role of Cys141 position in the AnGDH structure-activity relationship.

Interaction of 2-HED and AnGDH followed two distinct phases (Figure 5). The forward velocity, when monitored by the described standard assay, was selectively and rapidly lost in the first phase; the reverse activity was also affected upon prolonged incubation. The enzyme at the end of the first phase (referred to as ‘FIGDH’) behaves like a hypothetical ‘one-way active’ enzyme. The FIGDH (with <1.0% activity in the standard forward assay and almost 100% activity in the standard reverse assay, when compared to AnGDH) could be isolated by careful choice of 2-HED concentration and incubation time. Many enzymes have evolved to catalyze reactions preferentially in one direction by virtue of their unique kinetic parameters. While the Haldane relationship places limits on its kinetic features, an enzyme catalyst can still display a range of kinetic behavior [39]–[43]. One-way inhibition in the presence of effectors, offers ways to modulate the enzyme function as and when desired. Such unidirectional inhibition has attracted some attention and is reported for a few enzymes [12]–[17]. Except sucrose synthase [16] all others are examples of non-covalent enzyme-effector interactions. Although sucrose cleavage activity of sucrose synthase is inhibited (to about 70% of the control) by thiol modification, it is not as striking an example as the preferential attenuation of AnGDH reductive amination activity reported here.

The FIGDH form of the enzyme showed considerably altered kinetic properties namely, sigmoid l-glutamate (a reverse substrate) saturation and diminished apparent affinity towards the three forward substrates (Table 1). These affinity changes do contribute to the apparent unidirectional attenuation of FIGDH activity as measured in the standard assay. The lag observed in the time course of FIGDH forward reaction also implicates a role for enzyme conformational changes. It may be emphasized that the conditions under which the measurements were made are different for the two directions. The measured K_eq_ for the GDH reaction catalyzed by both AnGDH and FIGDH forms was identical (Figure 6) and comparable with the values reported earlier [27], [44], [45]. And the approach to equilibrium from the reverse direction is similar for both the enzyme forms. However, they behave quite differently in the forward direction. While the AnGDH curve is steep, the FIGDH response is much flatter reflecting a very sluggish approach to equilibrium. Although l-glutamate formation is thermodynamically favored, NAD-dependent GDHs are known to efficiently catalyze the oxidative deamination of glutamate [1]. In this sense, FIGDH resembles the NAD-dependent catabolic GDH.

The kinetics of inhibition of AnGDH forward activity by 2-HED (Figure 3) and DTNB titrations (Table 3) suggested that a single thiol modification results in FIGDH. This thiol was identified by site-directed mutagenesis. Only two (Cys141 and Cys415) of the five cysteines present in AnGDH were selected for site-directed mutagenesis based on homology modeling. The C141S mutant did not significantly differ in its physical and kinetic properties when compared with the native enzyme (Table 1). This mutant was however insensitive to 2-HED treatment (Figure 5) indicating that Cys141 modification occurred in FIGDH and is responsible for forward inhibition. Prior incubation of FIGDH with NADPH and 2-oxoglutarate abolished the lag in the forward initial velocity. Similar kinetic behavior was observed with a few *am* mutants of *Neurospora crassa* NADP-GDH [46]. Some of these were activated after pre-incubation with glutamate/succinate while others attained maximal reaction rate only after a lag period. Interestingly, many of these *am* mutations map to a region surrounding His141 residue (corresponding to Cys141 in AnGDH sequence).

Analysis of crystal structure data for AnGDH (at 2.5 Å resolution) shows presence of Cys141 in the interface region of the hexamer (personal communication; Dr. Prasenjit Bhaumik, Institute of Technology Bombay, Mumbai, India). While awaiting further structural information on AnGDH and other forms, we performed homology modeling of AnGDH to discern the environment around Cys141 and Cys415 (Figure 4). Cys141 is solvent exposed and is near to the inter-subunit interfaces. It is located in proximity (within 15 Å distance) to Lys78, Gly80, Lys114 and Ala152 – all are conserved residues in or near the glutamate-binding-pocket in other NAD(P)-GDHs [47]-. That the mixed disulfide of Cys141 (with –S-CH_2_-CH_2_-OH) notably alters the interaction with glutamate-binding-domain substrates is consistent with this geometry. Site-directed mutants with bulky side chains at position 141 could be explored to mimic FIGDH (C141K and C141M are being characterized for this purpose). Cys415 is not involved in the 2-HED dependent forward inhibition of AnGDH. But C415S mutation is interesting in that it leads to unusual kinetic features. C415S mutant has an increased *n*_H_ for 2-oxoglutarate saturation and its ammonium saturation is pronouncedly biphasic (to be communicated separately). In the homology model, Cys415 is closer (∼10 Å distance) to Lys102 – equivalent residue near the l-glutamate binding site in other GDHs studied [49], [53]. The two site-directed mutants, namely C141S and C415S, offer an excellent opportunity to study allosteric interactions in AnGDH. Crystal structures of various enzyme forms underway, including FIGDH, are expected to help correlate kinetic features with active site geometry.

Thiol-based regulation of enzyme activity in plants has received much attention; glutaredoxin, thioredoxin and cellular thiols are important in redox regulation of chloroplast metabolism [54]. The *A. niger* NADP-GDH activity is unaffected by hydrogen peroxide induced oxidative stress [55]. However, specific thiol-disulfide mediated effects on metabolism are not reported in this fungus. Reversible modulation observed with the cysteine/cystine redox pair suggests the feasibility of NADP-GDH regulation *in vivo*; this is yet to be tested. In summary, Cys141 was identified as the target for unidirectional attenuation of *A. niger* NADP-GDH by 2-HED. That the Cys141 modification switches the biosynthetic enzyme (AnGDH) into a catabolic one (FIGDH) is noteworthy.

## Supporting Information

Figure S1 **Comparison of physical properties of AnGDH, its two C→S mutants and FIGDH.** **A)** CD spectra of different AnGDH enzyme forms (at 2.7 µM) were recorded. Native PAGE of these proteins stained with Coomassie Blue R-250 (lane 1, FIGDH; lane 2, AnGDH; lane 3, C141S and lane 4, C415S) is shown as inset. **B)** Elution profiles of different GDH forms on HiLoad 16/60 Superdex 200 column. Their native molecular masses calculated from elution volume data is shown in the table (inset). Forward activity was monitored for AnGDH and C141S while FIGDH and C415S were monitored using reverse assay.
(TIF)

Figure S2 **pH optima for forward and reverse reactions catalyzed by AnGDH and FIGDH.** The GDH activity (•, AnGDH forward; ▴, FIGDH forward; ○, AnGDH reverse; Δ, FIGDH reverse) was measured in a combination buffer (containing 25 mM MES, 25 mM acetate and 50 mM Tris) adjusted to different pH values. FIGDH forward reaction was monitored at 370 nm in a reaction mixture containing 50 mM 2-oxoglutarate, 10 mM ammonium and 200 µM NADPH. The enzyme was pre-incubated with 2-oxoglutarate and NADPH for 15 min before starting the reaction (by ammonium). All the other activity measurements were according to the standard assay (see Materials and Methods).
(TIFF)
