# Supplementary figures and images for: Mixed Disulfide Formation at Cys141 Leads to Apparent Unidirectional Attenuation of Aspergillus niger NADP-Glutamate Dehydrogenase Activity
Source: PLoS One. 2014 Jul 2;9(7):e101662. doi: 10.1371/journal.pone.0101662 (PMC4079599; doi:10.1371/journal.pone.0101662)

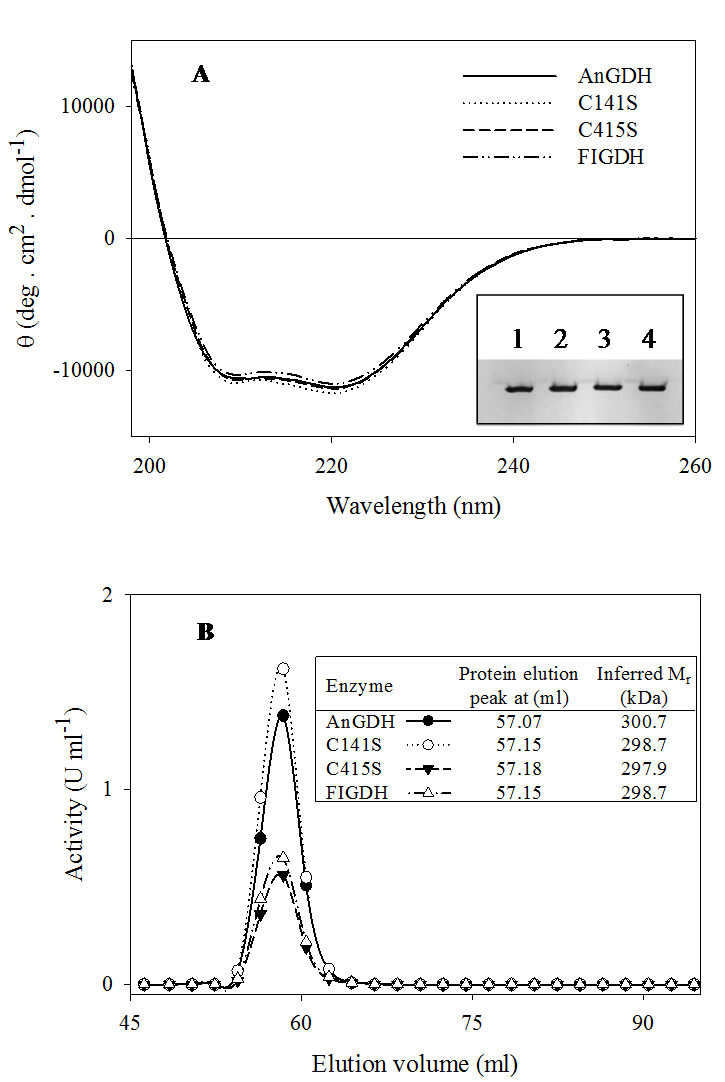

Supplement: Figure S1 — Comparison of physical properties of AnGDH, its two C→S mutants and FIGDH. A) CD spectra of different AnGDH enzyme forms (at 2.7 µM) were recorded. Native PAGE of these proteins stained with Coomassie Blue R-250 (lane 1, FIGDH; lane 2, AnGDH; lane 3, C141S and lane 4, C415S) is shown as inset. B) Elution profiles of different GDH forms on HiLoad 16/60 Superdex 200 column. Their native molecular masses calculated from elution volume data is shown in the table (inset). Forward activity was monitored for AnGDH and C141S while FIGDH and C415S were monitored using reverse assay. (TIF) [file pone.0101662.s001.tif]
